# Sex-specific genetic effects in physical activity: results from a quantitative genetic analysis

**DOI:** 10.1186/s12881-015-0207-9

**Published:** 2015-08-01

**Authors:** Vincent P. Diego, Raquel Nichele de Chaves, John Blangero, Michele Caroline de Souza, Daniel Santos, Thayse Natacha Gomes, Fernanda Karina dos Santos, Rui Garganta, Peter T. Katzmarzyk, José AR Maia

**Affiliations:** 1 University of Texas Rio Grande Valley, School of Medicine, South Texas Diabetes and Obesity Institute, Brownsville, Texas; 2 Academic Department of Physical Education, Federal University of Technology - Parana, Curitiba - PR, Brazil; 3 CIFI²D, Kinanthropometry Lab, Faculty of Sport, University of Porto, Porto, Portugal Faculty of Sports, University of Porto, Porto, Portugal; 4 Department of Physical Education and Sports Science, CAV, Federal University of Pernambuco, Vitória de Santo Antão, Brazil; 5 Pennington Biomedical Research Center, Baton Rouge, USA

**Keywords:** Sex effects, Heritability, Physical activity, Sedentary behaviour

## Abstract

**Background:**

The objective of this study is to present a model to estimate sex-specific genetic effects on physical activity (PA) levels and sedentary behaviour (SB) using three generation families.

**Methods:**

The sample consisted of 100 families covering three generations from Portugal. PA and SB were assessed via the International Physical Activity Questionnaire short form (IPAQ-SF). Sex-specific effects were assessed by genotype-by-sex interaction (GSI) models and sex-specific heritabilities. GSI effects and heterogeneity were tested in the residual environmental variance. SPSS 17 and SOLAR v. 4.1 were used in all computations.

**Results:**

The genetic component for PA and SB domains varied from low to moderate (11 % to 46 %), when analyzing both genders combined. We found GSI effects for vigorous PA (p = 0.02) and time spent watching television (WT) (p < 0.001) that showed significantly higher additive genetic variance estimates in males. The heterogeneity in the residual environmental variance was significant for moderate PA (p = 0.02), vigorous PA (p = 0.006) and total PA (p = 0.001). Sex-specific heritability estimates were significantly higher in males only for WT, with a male-to-female difference in heritability of 42.5 (95 % confidence interval: 6.4, 70.4).

**Conclusions:**

Low to moderate genetic effects on PA and SB traits were found. Results from the GSI model show that there are sex-specific effects in two phenotypes, VPA and WT with a stronger genetic influence in males.

## Background

It has been widely observed that moderate to high physical activity (PA) levels are associated with lower risks for several chronic diseases, especially the major consequences of the metabolic syndrome, namely obesity, type 2 diabetes, and cardiovascular disease [1–3]. In spite of these observations, individuals in the general population tend to adopt less active or sedentary lifestyles [4–6]. A recent report showed that approximately 31.1 % of adults worldwide are physically inactive, with values ranging from 17 % (in southeast Asia) to about 43 % (America and eastern Mediterranean). Furthermore, approximately 80 % of adolescents between 13–15 years old do not reach the recommended daily PA values (60 min/day of moderate to vigorous PA) [7]. Moreover, there is a marked sex-difference in PA levels (regardless of how PA levels are measured), with males consistently having higher PA levels than females [4–8].

Here we treat sex as a composite internal environment [9, 10], and we ask if the sex environment has a significant effect on the genetic variation underlying PA levels. Although results on the genetics of PA have increased over the last decade [11–15], there is still little known about how sex affects the genetic determinants of different expressions of PA phenotypes [16]. Previous studies that employed family designs in the genetic analysis of PA levels have demonstrated that genetic effects can account for a significant proportion (as high as 60 %) of PA variability [17–21]. However, such studies did not test for possible genotype-by-sex interaction (GSI) underlying sex-specific effects in PA. One widely used approach towards addressing this issue is to make inferences on the basis of sex-specific heritabilities of PA. As we demonstrate in this report, however, sex-specific heritability is a potentially misleading measure of the importance of sex-specific genetic effects, and can in fact lead to inaccurate inferences. To see why sex-specific heritability is potentially misleading in regard to sex-specific genetic effects, consider the definition of the heritability as the ratio of the additive genetic variance to the total phenotypic variance, which in the absence of genetic marker data is simply the sum of the additive genetic and residual environmental variances. Because the heritability is a ratio, its value can change as a result of changes in the genetic effects or in the residual environmental effects, or in both. Thus, with respect to the objective of elucidating sex-specific genetic effects per se, sex-specific heritability estimates can be indecisive and/or misleading . Alternatively,, we advocate a formal GSI model [22, 23] designed to detect sex-specific genetic effects *sensu strictu* in PA. We contrast this formal GSI model with a re-parameterized form of the model designed to detect sex-specific heritabilities in PA. In contrasting the two, we show that whereas the formal GSI model can efficiently detect sex-specific genetic effects, the sex-specific heritability model can and does lead to improper inferences. Thus, the primary aim of this study is to investigate potential sex-specific genetic effects influencing PA levels and sedentary behaviour (SB), and the secondary aim is to show that GSI modelling and sex-specific heritability modelling can lead to different conclusions.

## Methods

### Sample

The study used cross-sectional PA and SB data arising from three generation families comprising 1034 subjects from 100 families (517 females and 517 males), aged 7 to 85 years old from Portugal. The University of Porto Ethics committee approved the study and written informed consent was obtained from all subjects. The parents or legal guardian signed the written informed consent of the children and adolescents. The average family size was 10, with a range of 9 to 16 subjects. Due to missing data, only 724 individuals from 100 families were included in the analysis.

### Physical activity and sedentary behaviour

PA and SB were measured with the International Physical Activity Questionnaire, short form (IPAQ-SF). This instrument has acceptable measurement properties in estimating PA levels with international validation results previously reported [8, 24].

Participants aged below 15 years were interviewed by a team previously trained according to the standardized IPAQ-SF approach. All other individuals self-reported their responses to all questionnaire items. A random sample of 10 families was retested two weeks apart and reliability values (ANOVA-based intraclass correlation coefficient) within each family member were around 0.80.

Participants answered about intensity, frequency and duration of their physical activities during one week, as well as about their SB. Derived phenotypes were: vigorous PA (VPA); moderate PA (MPA); walking (WK); total PA (TPA); time spent watching television (WT) and sitting time (ST). Standard procedures were adopted, using continuous scales of weekly energy expenditure to represent several PA levels, expressed by metabolic equivalent (MET/minutes/week). The reference values for the calculation of energy expenditure were: VPA = 8.0 METs; MPA = 4.0 MET’s; and WK = 3.3 MET’s. TPA is an index of the sum of the values reported for VPA, MPA and WK. SB values were maintained in minutes per day, according to the IPAQ protocol [25].

### Statistical analysis

SPSS version 17.0 was used for exploratory data analysis in order to verify probable data entry errors as well as the presence of outliers*.* Due to high skewness and kurtosis, median values and interquartile range were used to describe all phenotypes. PA and SB phenotypes were adjusted for the following covariate effects: age, sex, age^2^, sex*age, sex*age^2^. The residuals obtained after accounting for the covariate effects were then transformed using an inverse normal transformation [26, 27].

We now describe the statistical genetic models employed in our analyses. Assuming that dominance and epistasis are negligible, comparisons of individual phenotype *y* define the covariance of the basic polygenic model as:

1 $$ Cov\left({y}_x,{y}_z\right)=2{\phi}_{xz}{\sigma}_g^2+{\sigma}_e^2{\delta}_{xz} $$

where *x* and *z* index individuals, 2*ϕ*_*xz*_ gives the expected coefficient of relationship, *σ*_*g*_^2^ is the additive genetic variance, *σ*_*e*_^2^ is the environmental variance, and *δ*_*xz*_ is defined as 1 when individuals *x* and *z* are the same and 0 otherwise. This model is used to estimate heritability, which is given as: ${h}^2=\frac{\sigma_g^2}{\sigma_g^2+{\sigma}_e^2}=\frac{\sigma_g^2}{\sigma_p^2}$, where *σ*_*p*_^2^ is the total phenotypic variance. The model can be extended to account for GSI effects by allowing for sex-specific additive genetic and environmental variances denoted by *σ*_*gf*_^2^, *σ*_*gm*_^2^, *σ*_*ef*_^2^, and *σ*_*em*_^2^, where *f* and *m* index females and males, respectively, and for an across-sex genetic correlation denoted by *ρ*_*g*(*f*,*m*)_.

In principle, sex-specific heritabilities can be computed from the relevant parameter estimates under this GSI model. However, for formal statistical inferences under the model, we would have no recourse but to use crude approximations of the standard errors for the sex-specific heritabilities, and an associated Wald test statistic. Worse still, the Wald test statistic formed in this case from variance component estimates is known to be problematic [28, 29]. Therefore, in order to be able to estimate sex-specific heritabilities for which reliable statistical inferences can be made, we developed a reparameterized version of the GSI model by instead allowing for sex-specific phenotypic variances, heritabilities, and corresponding "environmentalities", respectively denoted by *σ*_*pf*_^2^, *σ*_*pm*_^2^, *h*_*f*_^2^, *h*_*m*_^2^, (1 − *h*_*f*_^2^) = *e*_*f*_^2^, and (1 − *h*_*m*_^2^) = *e*_*m*_^2^, while maintaining the same parameterization for the across-sex genetic correlation.

On finding the maximum likelihood estimates (MLEs) of the parameters, defined as estimates that make the full likelihood function a maximum, and the model likelihoods under the null and alternative hypotheses, inferences are then made by way of the likelihood ratio test and its associated test statistic (LRT). The LRT is computed as minus twice the difference in ln-likelihoods estimated under the null and alternative hypotheses, and is in standard cases distributed as a chi-square with degrees of freedom (d.f.) given by the difference in the number of estimated or unconstrained parameters. However, as is often the case for variance components models, the non-standard case of testing a null hypothesis lying at a boundary of its acceptable parameter range yields a LRT distributed as a 50:50 mixture of a point-mass at 0 and a chi-square with 1 d.f., which we write as $\left({\scriptscriptstyle \frac{1}{2}}{\chi}_0^2+{\scriptscriptstyle \frac{1}{2}}{\chi}_1^2\right)$ [30, 31]. For example, the null hypothesis that the heritability equals zero lies at a boundary of its acceptable parameter range. In this case, the LRT is distributed as $\left({\scriptscriptstyle \frac{1}{2}}{\chi}_0^2+{\scriptscriptstyle \frac{1}{2}}{\chi}_1^2\right)$*.*

In this report we are chiefly concerned with addressing the existence of GSI effects and of sex-specific heritabilities. It can be shown that in order for there to be a GSI effect, there must be either heterogeneity in the additive genetic variance across environments *(i.e. σ*_*gf*_^2^ ≠ *σ*_*gm*_^2^*)* or the genetic correlation across environments must be different from unity *(i.e. ρ*_*g*(*f*,*m*)_ ≠ 1*)* or both. Thus, for GSI we have two null hypotheses to test, namely *σ*_*gf*_^2^ = *σ*_*gm*_^2^ = *σ*_*g*_^2^ and *ρ*_*g*(*f*,*m*)_ = 1*.* When testing *σ*_*gm*_^2^ = *σ*_*gf*_^2^ = *σ*_*g*_^2^*,* we have a 1 d.f. difference between models compared, and so the LRT is distributed as *χ*_1_^2^*.* When testing *ρ*_*g*(*f*,*m*)_ = 1*,* we again have a 1 d.f. difference, but the relevant null hypothesis, namely *ρ*_*g*(*f*,*m*)_ = 1*,* lies at a boundary of its acceptable parameter range, and so the LRT in this case is distributed as $\left({\scriptscriptstyle \frac{1}{2}}{\chi}_0^2+{\scriptscriptstyle \frac{1}{2}}{\chi}_1^2\right)$*.* Using the GSI model, we can also test the null hypothesis that the sex-specific residual environmental variances are equal, namely *σ*_*ef*_^2^ = *σ*_*em*_^2^ = *σ*_*e*_^2^*.* For this case, the LRT is distributed as *χ*_1_^2^*.* The next inferential step is to test if each of the sex-specific heritabilities are significantly different from 0, with LRTs distributed as $\left({\scriptscriptstyle \frac{1}{2}}{\chi}_0^2+{\scriptscriptstyle \frac{1}{2}}{\chi}_1^2\right)$*.*

Reports in the literature of sex-specific heritabilities are invariably presented as interval estimates. In order to make our results comparable with the previous studies, we constructed likelihood-based 95 % confidence intervals (CIs) following recommendations in the literature [32, 33]. A likelihood-based 95 % CI around a given maximum likelihood estimate (MLE) is one that satisfies the following general formula:

2 $$ \mathsf{L}\mathsf{R}\mathsf{T}\le {\chi}_{\nu}^2\left(\alpha \right). $$

where the LRT is computed by comparing a model for a given MLE effect size (ES) to a model with a parameter ES constrained in the direction of the lower or upper bound that satisfies the formula, and *χ*_*ν*_^2^(*α*) is the chi-square with the relevant d.f. under the comparison which corresponds to the nominal level of significance given by *α*, which in our case is *α* = 0.05. Thus, to compute the 95 % CI with respect to the null hypothesis of equality of sex-specific heritabilities or that a given sex-specific heritability is different from zero we find the parameter estimates satisfying:

3 $$ \mathsf{L}\mathsf{R}\mathsf{T}\le {\chi}_1^2\left(\alpha =0.05\right)\approx 3.84 $$

In one case in the ensuing it was not possible to compute a 95 % CI using this approach. Thus, we used the following formula for an approximate 95 % CI [34], where SE is the standard error:

4 $$ 95\%\ \mathsf{C}\mathsf{I}=\mathsf{E}\mathsf{S}\pm 1.96\times \mathsf{S}\mathsf{E} $$

To help shed light on our findings, we peformed a post-hoc power analysis. Power is defined as the probability of correctly rejecting the null hypothesis, and is computed as the probability integral from the point on the alternative distribution corresponding to the nominal significance level or alpha (on the null distribution) to the upper limit of the alternative distribution at positive infinity. Since the total probability of any distribution is 1, power can be conveniently computed as:

5 $$ \begin{array}{l} \Pr \left({\sigma}_{gf}^2\ne {\sigma}_{gm}^2\right)\ \mathrm{or}\  \Pr \left({\rho}_{g\left(f,m\right)}<1\right)={\displaystyle \underset{\chi_{\alpha; \nu, \xi =0}^2}{\overset{\infty }{\int }}d{\chi}_{\nu, \xi}^2}={\displaystyle \underset{0}{\overset{\infty }{\int }}d{\chi}_{\nu, \xi}^2}-{\displaystyle \underset{0}{\overset{\chi_{\alpha; \nu, \xi =0}^2}{\int }}d{\chi}_{\nu, \xi}^2}\\ {}\\ {}\kern13em =1-{\displaystyle \underset{0}{\overset{\chi_{\alpha; \nu, \xi =0}^2}{\int }}d{\chi}_{\nu, \xi}^2}=1-\beta, \end{array} $$

where the distribution under the alternative hypothesis is the non-central chi-square distribution, denoted by *χ*_*ν*,*ξ*_^2^, *ν* is the degrees of freedom (d.f.) parameter, *ξ* is the non-centrality parameter (NCP), *χ*_*α*;*ν*,*ξ* = 0_^2^ is the point on the non-central chi-square distribution corresponding to the 100(1 − *α*) percentage point on the distribution under the null hypothesis, and $\beta ={\displaystyle \underset{0}{\overset{\chi_{\alpha; \nu, \xi =0}^2}{\int }}d{\chi}_{\nu, \xi}^2}$ is the probability of making a type II error. We used a similar approach to that used by Blangero et al. [35].

## Results and discussion

Main descriptive statistics for all PA and SB phenotypes are presented in Table 1. About half of first and second generation subjects did not report VPA. The TPA levels increase across generations. Reported SB has higher values in the first generation, as expected, although second and third generation subjects had similar values.

**Table 1:** Descriptive statistics for age, physical activity levels and sedentary behaviour by generation and sex

		1st Generation		2nd Generation		3rd Generation	
		Grandfather	Grandmother	Father	Mother	Son	Daughter
		*n = 104*	*n = 104*	*n = 147*	*n = 147*	*n = 111*	*n = 111*
VARIABLES							
		Mean ± sd		Mean ± sd		Mean ± sd	
Age (years)		74.4 ± 10.7	76.4 ± 7.6	41.9 ± 5.2	40.9 ± 4.9	13.6 ± 2,4	13.3 ± 2.5
		Median (IQR)		Median (IQR)		Median (IQR)	
VPA (mets/min/week)		0 (0)	0 (0)	0 (320)	0 (160)	280 (960)	80 (640)
MPA (mets/min/week)		0 (80)	0 (0)	140 (400)	160 (360)	280 (620)	240 (480)
WK (mets/min/week)		330 (297)	247,5 (256)	743 (660)	660 (561)	718 (1068)	743 (990)
SB	ST (min/day)	420 (180)	420 (120)	300 (120)	300 (120)	360 (120)	360 (120)
	WT (min/day)	155 (138)	180 (120)	80 (75)	80 (60)	92,5 (80)	105 (60)
TPA (mets/min/week)		413 (291)	264 (346)	1058 (923)	1015 (913)	1722,25 (1571)	1545 (1435)

sd: standard deviation; IQR: interquartile range; VPA: vigorous physical activity; MPA: moderate physical activity; WK: walking; SB: sedentary behaviour; ST: sitting time; WT: watching television; TPA: total physical activity

Heritability estimates for the five phenotypes are presented in Table 2. All traits are statistically significant ranging from 0.112 (WT) to 0.456 (WK). The covariates explained 2 % to 30 % of the phenotypic variance.

**Table 2:** Heritability estimates (h^2^), standard errors (se), p-values and explained variance by covariates

Trait	h^2^ ± se	p-value	Variance explained by covariates (%)^a^
VPA	0.280 ± 0.061	1.0 × 10^−7^	2.0
MPA	0.312 ± 0.060	2.4 × 10^−9^	8.0
WK	0.456 ± 0.058	3.1 × 10^−18^	15.0
ST	0.287 ± 0.059	4.1 × 10^−8^	16.0
WT	0.112 ± 0.061	2.5 × 10^−2^	30.0
TPA	0.284 ± 0.059	3.4 × 10^−8^	10.0

VPA: vigorous physical activity; MPA: moderate physical activity; WK: walking; ST: sitting time; WT: watching television; TPA: total physical activity. Covariates in the regression models included age, sex, age^2^, sex*age, sex*age^2^

**Fig. 1:**
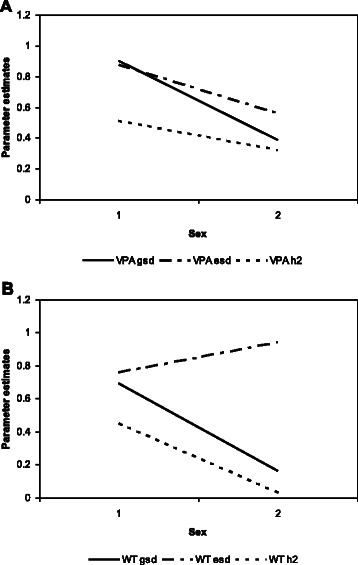
Genotype × sex interaction effects and sex-specific heritabilities for VPA and WT. **a**. VPA. **b**. WT. Parameter estimates on the vertical axis plotted against sex on the horizontal axis. Male and female sexes are coded respectively as 1 and 2. Additive genetic and environmental standard deviations (gsd and esd, respectively) are respectively given by the solid and dot-dashed lines, and sex-specific heritabilities (h2) are given by the dotted lines

We found GSI effects for VPA (p = 0.0005) and WT (p = 0.02), and heterogeneity in the residual environmental variance for VPA (p = 0.006), MPA (p = 0.02) and TPA (p = 0.001) (Table 3). Sex-specific heritability estimates are reported in Table 4, showing significantly higher values in males for WT but not for VPA (Fig. 1).

**Table 3:** P-values for genotype-by-sex interaction effects

Null hypothesis test:			
Trait	Equal sex-specific additive genetic variances	Genetic correlation coefficient equal to 1	Equal sex-specific residual environmental variances
VPA	0.0005	0.08	0.006
MPA	0.33	0.18	0.02
WK	0.78	0.36	0.27
ST	0.27	0.50	0.65
WT	0.02	0.23	0.10
TPA	0.24	0.20	0.001

VPA: vigorous physical activity; MPA: moderate physical activity; WK: walking; ST: sitting time; WT: watching television; TPA: total physical activity

**Table 4:** Sex-specific heritabilities

Trait	% h^2^ (95 % CI)	% h^2^ (95 % CI)	Δh^2^ (95 % CI)^a^
	Males	Females	
VPA	51.364 (29.75, 72.25)	32.004 (9.8, 55.59)	19.3 (−13.4, 50.7)
MPA	35.477 (13.3, 58.6)	39.938 (18.4, 62)	−4.461 (−36.1, 27.2)
TPA	32.931 (12.28, 54.4)	37.909 (17.03, 59.44)	−4.978 (−35.8, 25.6)
WT	45.293 (18.9, 72.25)	2.8 (−20.916, 26.516)	42.485 (6.4, 70.4)

^a^Δh^2^ = male % h^2^ – female % h^2^. The null hypothesis that Δh^2^ = 0 is equivalent to the null hypothesis that the sex-specific heritabilities are equal

VPA: vigorous physical activity; MPA: moderate physical activity; WK: walking; ST: sitting time; TPA: total physical activity; WT: watching television

We report power to detect GSI effects for each trait in Table 5. There was sufficient power to detect GSI due to heterogeneity in the additive genetic variance for only VPA but not for the other traits. Also, there was sufficient power to detect GSI due to a genetic correlation coefficient less than 1 for only WT but not for the other traits. That we found significant GSI due to additive genetic variance heterogeneity in VPA is not too surprising given that we had sufficient power for this trait. On the other hand, there are two related issues regarding the power analysis results for WT that need some explanation. The first issue is that we found significant GSI due to additive genetic variance heterogeneity for WT but the power to detect this GSI effect was not sufficient. The second issue is the converse in that we had maximum power to detect a GSI effect due to a genetic correlation less than 1 and yet we failed to reject the null hypothesis of *ρ*_*g*(*f*,*m*)_ = 1. On these issues, we note the following observations. Low power does not entirely preclude the rejection of a null hypothesis. Power merely measures the probability at which rejection of a false null hypothesis may occur. Thus, it is possible (though perhaps not so probable) to have low power and yet reject a false null hypothesis. Conversely, maximum power simply means that if a null hypothesis is false, then it will be rejected every time, but it does not necessarily mean that the null hypothesis is in fact false. It is well known that complex morphological and physiological traits may often exhibit marked sexual dimorphism, especially traits associated with body fat distribution and physiological weight regulation [36–38]. Marked sex-specific variation is also evident in behavioral characteristics such as PA and SB, and may have a significant genetic component or may be modulated by genetic determinants through sex-specific effects [16]. To assess these sex-specific effects on several PA and SB phenotypes, we employed models of GSI effects and of sex-specific heritabilities. We tested for GSI effects and heterogeneity in the residual environmental variance. Then, to be able to compare our results with reports in the literature, which are usually framed in terms of sex-specific heritabilities, we tested for sex-specific heritabilities in our data.

**Table 5:** Statistical power analysis for the trait-specific parameters

Trait	Power or probability of rejecting a false null hypothesis for the following null hypotheses for genotype × sex interaction	
	*σ*_*gf*_^2^ = *σ*_*gm*_^2^ = *σ*_*g*_^2^	*ρ*_*g*(*f*,*m*)_ = 1
VPA	0.93558	0.39621
MPA	0.16386	0.24019
WK	0.05925	0.12345
ST	0.19644	0.1
WT	0.67527	1
TPA	0.22014	0.22074

**Table 6:** Sex-specific heritabilities of different physical activity phenotypes in twin studies

Author	Ano	Country	Trait	% h^2^ (95 % CI)	% h^2^ (95 % CI)
				Males	Females
Boomsma et al. [55]	1989	Netherlands	SP	77 (NA)	35 (NA)
Beunen & Thomis [51]	1999	Belgium	SP	44 (21–91)	83 (66–91)
Maia et al. [52]	2002	Portugal	LTPA	66 (48.9-73.3)	32 (0.4-61.8)
Maia et al. [52]	2002	Portugal	SPI	68.4 (41.5-89.2)	39.8 (0.4-73.0)
Carlsson et al.[56]	2006	Sweden	LTPA	64 (55–72)	51 (48–60)
		(14–28 yrs)			
Carlsson et al.[56]	2006	Sweden	LTPA	40 (30–45)	41 (30–45)
		(29–46 yrs)			
Stubbe et al. [57]	2006	Australia	EP	22.9 (0–56.1)	31.1 (0.3-55.6)
Stubbe et al.[57]	2006	Denmark	EP	44 (24.2- 55.7)	50.1 (30.3-57.7)
Stubbe et al.[57]	2006	Finland	EP	55.8 (38.4-63.3)	61 (44.5-66.3)
Stubbe et al.[57]	2006	Netherlands	EP	68.1 (34.2-79.0)	50.3 (21.3-70.3)
Stubbe et al.[57]	2006	Norway	EP	33.6 (6.7-61.7)	56.6 (46.5-63.8)
Stubbe et al.[57]	2006	Sweden	EP	63.9 (52.1-68.6)	59.5 (46.9-64.7)
Aaltonen et al.[50]	2010	Finland	LTPA	53 (44–60)	38 (28–40)

h^2^: heritability; CI: confidence intervals; SP: sport participation; LTPA: leisure-time physical activity; SPI: sport participation index; EP: exercise participation

In the present study, low to moderate genetic contributions to PA and SB phenotypes were found, ranging from 11 % to 46 %. These results are in accord with previous quantitative genetic studies on nuclear or extended families, which have suggested considerable genetic influence in these traits, notwithstanding the methodological differences to measure PA and SB traits and the diversity in statistical procedures [39]. It should be noted, however, that studies in monozygotic and dizygotic twins have reported greater intraclass correlations for PA levels suggesting moderate to high genetic influences, from 13 % to 98 % [39].

We found evidence of significant GSI for VPA and WT. In particular, VPA and WT exhibited GSI via significant heterogeneity in the additive genetic variance across genders. For VPA and WT, the additive genetic variance was higher in males than in females. Further, evidence of significant heterogeneity in the residual environmental variance for WT, but not for VPA was shown. Our analysis shows that the male-specific heritabilities for VPA and WT and the female-specific heritability for VPA were significantly different from zero. As can be expected from the GSI results, the male- and female-specific heritabilities for WT were significantly different from each other, which is consistent with Brazilian families’ sedentarism trait shown by Horimoto et al. [16], although their models were parameterized differently, considered no covariates, and had a distinct definition of sedentarism. Surprisingly, however, we found that the male- and female-specific heritabilities for VPA were not significantly different from each other. The explanation for this apparent discrepancy between the GSI and sex-specific results for VPA is actually quite simple. For VPA, the difference across the sexes in the residual environmental variance was of similar magnitude and direction as the difference across genders in the additive genetic variance, and thus the sex-specific heritabilities—recall that heritability is the proportion of the additive genetic variance to the total phenotypic variance—were quite similar for both males and females. For WT, the additive genetic variance was significantly different across sexes but not the residual environmental variance. Consequently, the sex-specific heritability for WT was significantly lower in females compared to males, which is in opposition to the Brazilian data from Horomito et al. [16], in which the heritability was 22 % for females and 5 % for males. It is important to stress that in their study, if a family member did not take part in sports, they were classified as having a sedentarism phenotype. Furthermore, in their analysis no heterogeneity in variance by gender was found when they adjusted their models to age, sex and age-by-sex interaction covariates. Variance heterogeneity was only found when no covariates were included in the models.

Most studies on the relationship between PA levels and WT-like measures have found that the two variables tend to show either a weak relationship or no relationship at all [40–44]. Further, at least two studies have shown that PA levels and WT-like variables have independent effects on cardiovascular disease risk factors, which seems to suggest that there is a weak or even no relationship between the two variables [45, 46]. We should point out, however, that a few studies have found an inverse relationship between PA levels and WT-like measures [47–49]. Nonetheless, evidence from the majority of studies conversant on this issue supports the hypothesis that PA levels and WT-like measures are only weakly associated at best.

Our results introduce an interesting twist to this weak relationship between PA levels and WT-like measures. We showed that the residual environmental variance decreased from males to females for VPA but not for WT. A plausible two-fold explanation of these different patterns is given as follows. It may be that for whatever set of sociocultural reasons there are more opportunities for PA for males than for females (e.g. leisure time practice of team sports) and/or a greater emphasis placed on males relative to that placed on females to be physically active. Additionally, to explain the effectively similar levels of residual environmental variance in males and females for WT it may be that there is enough of a variety of TV shows to equally attract the attention of both sexes and/or that there are TV shows both sexes tend to watch. In the VPA case, the sex-specific heritabilities were rendered statistically indistinguishable by virtue of the residual environmental variance, even in the face of significant heterogeneity in the sex-specific additive genetic variance. These results serve as a sobering reminder that heritabilities are also significantly influenced by the residual environmental variance.

A number of twin studies have reported sex-specific heritabilities of several PA traits, including leisure time PA (LTPA), sports participation (SP), and SP index (SPI) (Table 6). As can be gathered from Table 6, the 95 % CIs on the heritability estimates overlap, which implies that the sex-specific heritabilities are not different. However, as we showed for VPA it is quite possible that there is significant GSI even in the face of effectively equal sex-specific heritabilities. Moreover, although we did not find evidence of the across-sex genetic correlation being different from one, it is in theory possible for the across-sex genetic correlation to be different than one in these studies. Thus, it may still be possible that, even though most of the studies in Table 6 report sex-specific heritabilities that are not significantly different from each other, there are still significant GSI effects influencing these traits. Conversely, our results logically imply that even if sex-specific heritabilities are significantly different, as in the study by Aaltonen et al. [50] in Table 6, it may not be due to heterogeneity in the additive genetic variance but instead to heterogeneity in the residual environmental variance. That is, it is possible that the sex-specific heritabilities are rendered statistically dissimilar by virtue of heterogeneity in the residual environmental variance, even in the face of an additive genetic variance that is stable across the sexes. These considerations show that in order to be able to make robust statistical inferences about sex-specific genetic determinants it is not enough to estimate sex-specific heritabilities, but rather a formal GSI model must be employed.

The GSI model showed the presence of sex-specific effects in two phenotypes, namely VPA and WT. In light of the power analysis results, these findings are somewhat surprising in that our sample had low power overall to detect GSI effects. Comparing the present results with previous studies is difficult because we could only find one report from a similar extended family design [16], which, as previously mentioned, used a different set of PA phenotypes.. Studies with nuclear or extended families [19–21] typically just report mean values of different PA levels and tend to conclude that males are more active than females. However, GSI results are available from twin data analysis [50–52] and suggest the presence of a stronger genetic component in males than in females in some PA phenotypes. This dissimilarity between sexes may not be accurately explained by different sets of genes acting in males and females, which is consistent with our inability to reject the hypothesis that genetic correlation across the sexes is unity. However, it suggests a stronger genetic influence mainly in males. Besides a genetic predisposition towards being physically active or sedentary, genetic variation in PA levels may also be influenced by differences in body size and shape, motor performance, and psychological-drive to be physically active [51, 52].

Molecular genetic studies on PA levels and SB are scant, with little to offer in regard to sex-specific effects. The only association study reporting on this issue was by Simonen et al. [53] who found a consistent association between DNA sequence variation in the dopamine D2 receptor gene (DRD2) with PA levels only in females. To explain this finding they suggested that, because maternal-offspring correlations were consistently higher than paternal-offspring correlations, maternal genotype may have had a stronger impact on offspring’s PA levels relative to the effect of paternal genotype. Alternatively, they suggested that the effect of DRD2 in males could have been diluted by a stronger influence of environmental factors relative to that influencing females.

The results and conclusions of the present study may be affected by two main limiting factors. The first refers to the PA assessment through a self-administered questionnaire for individuals over 15 years-old. However, the International Physical Activity Questionnaire’s short form has acceptable measurement properties to access PA levels with validation in several countries [8, 24]. The second limitation has to do with inadequately accounting for household effects. In the case of the Brazilian family data previously mentioned, no significant household effects were found in any PA phenotype. Controlling for the shared environmental effects has been a difficult task. These involve several non-measurable living aspects within families and data regarding their magnitude are still inconsistent [17, 18, 21].

Although there are limitations, this study presents strengths to be highlighted. The participation of a large sample size and age range as well as relationship among different kinship degrees allow structuring expected covariates to a polygenic model [54]. The adequacy of our sample size in regard to the polygenic model notwithstanding, our power analysis results revealed that overall our study had low power to detect GSI effects. We can increase our power in this respect by increasing our sample size for future investigations and by using more sensitive PA and SB measures.

## Conclusions

In summary, our findings showed low to moderate genetic effects on PA and SB. Results from the GSI model show that there are sex-specific effects in two phenotypes, VPA and WT with a stronger genetic influence in males. This is consistent with reports in the literature suggesting a higher genetic component in males than females. We also found that inferences based on a GSI model can differ from those based on a sex-specific heritability model. Indeed, if our inferences were based solely on the latter approach we might have concluded that there are no statistically significant sex-specific genetic effects influencing VPA. This highlights the danger of relying on comparisons of sex-specific heritabilities to make such inferences. The present results advance our understanding of the genetic determinants of PA levels and SB traits. We are optimistic that studiesalong these lines will one day elucidate the presence of distinct PA- and SB-related genes in males versus females.

## Abbreviations

PA: Physical activity
GSI: Genotype-by-sex interaction
SB: Sedentary behaviour
IPAQ-SF: International Physical Activity Questionnaire short form
VPA: Vigorous physical activity
MPA: Moderate physical activity
WK: Walking
TPA: Total physical activity
WT: Time spent watching television
ST: Sitting time
MLEs: The maximum likelihood estimates
LRT: Likelihood ratio test
d.f.: Degrees of freedom
CI: Confidence interval
